# Multipotent ability of the baseline C-reactive protein-to-serum Albumin Ratio (CAR) to assess current activity and predict subsequent complications of ANCA-associated vasculitis

**DOI:** 10.1016/j.clinsp.2026.101217

**Published:** 2026-11-05

**Authors:** Hyesung So, Oh Chan Kwon, Jihye Chung, Lucy Eunju Lee, Jang Woo Ha, Yong-Beom Park, Sang-Won Lee

**Affiliations:** a Division of Rheumatology, Department of Internal Medicine, Yonsei University College of Medicine, Seoul, Republic of Korea; b Division of Rheumatology, Department of Internal Medicine, Gangnam Severance Hospital, Yonsei University College of Medicine, Seoul, Republic of Korea; c Division of Rheumatology, Department of Internal Medicine, Yongin Severance Hospital, Yonsei University College of Medicine, Yongin, Gyeonggi-do, Republic of Korea; d Institute for Immunology and Immunological Diseases, Yonsei University College of Medicine, Seoul, Republic of Korea

**Keywords:** Antineutrophil cytoplasmic antibody-associated vasculitis, C-Reactive protein, Albumin, Ratio, Prognosis

## Abstract

**Introduction:**

To investigate the ability of the baseline C-reactive protein-to-serum Albumin Ratio (CAR) to assess current vasculitis activity and predict subsequent complications in patients with antineutrophil cytoplasmic Antibody-Associated Vasculitis (AAV).

**Methods:**

Data from the medical records of 319 patients with AAV were retrospectively reviewed. High activity was defined as the highest tertile of the Birmingham Vasculitis Activity Score (BVAS). All-Cause Mortality (ACM), relapse, End-Stage Kidney Disease (ESKD), Cerebrovascular Accident (CVA), and Acute Coronary Syndrome (ACS) were evaluated as subsequent complications. ACM, ESKD, and CVA with significant Areas Under the Curve (AUC) in receiver operating characteristic curve analysis, were investigated.

**Results:**

The median age of the study subjects (64.6% females) was 60.0-years. Baseline CAR was independently correlated with current BVAS and yielded the numerically largest AUC for high activity. Patients with baseline CAR ≥ 3.07, ≥ 1.26, and ≥ 14.01 exhibited significantly higher Relative Risks (RR) for ACM (RR = 8.017), ESKD (RR = 3.270), and CVA (RR = 6.660) than those with baseline CAR < 3.07, < 1.26, and < 14.01, respectively. Additionally, patients with baseline CAR ≥ 3.07, ≥ 1.26, and ≥ 14.01 showed reduced cumulative patient, ESKD-free, and CVA-free survival rates compared with those with baseline CAR < 3.07, < 1.26, and < 14.01, respectively. Multivariable Cox proportional analysis revealed that baseline CAR and its complication-related cut-offs were independently associated with subsequent ACM, ESKD, and CVA, except for baseline CAR ≥ 1.26 for ESKD.

**Conclusions:**

Baseline CAR demonstrated the multipotent ability to reflect current high activity (the highest tertile of BVAS) and predict subsequent ACM, ESKD, and CVA in patients with AAV.

## Highlights

• Baseline CAR could reflect current activity, particularly high activity (the highest tertile of BVAS) in patients with AAV.
• Baseline CAR could predict subsequent ACM, ESKD, and CVA in patients with AAV.
• Accordingly, baseline CAR demonstrated the multipotent clinical utility in assessing current activity and predicting the prognosis of AAV.

## Introduction

Antineutrophil Cytoplasmic Antibody (ANCA)-Associated Vasculitis (AAV) is a small-vessel vasculitis, characterised by fibrinoid necrotising vasculitis involving mainly the capillaries, arterioles, venules, and occasionally medium-sized arteries.^1^ Because AAV can affect virtually all major organs, subsequent poor outcomes may present diverse manifestations, and severity varies from relapses to major-organ failure, which may result in serious complications such as All-Cause Mortality (ACM), relapses, End-Stage Kidney Disease (ESKD), Cerebrovascular Accident (CVA), and Acute Coronary Syndrome (ACS).^2^, ^3^, ^4^ Therefore, identifying baseline clues that predict subsequent serious complications of AAV during follow-up has clinical implications, as it could mitigate poor outcomes and improve the disease course. However, few baseline predictors of subsequent serious complications have been fully established to date, necessitating continued efforts to mine and discover novel baseline prognostic predictors.

The C-reactive protein (CRP)-to-serum albumin ratio (CAR) has been reported to predict the prognosis of various chronic inflammatory, cardiovascular and cancerous diseases.^5^, ^6^, ^7^, ^8^ Additionally, we have previously demonstrated the clinical significance of baseline CAR as an independent predictor of ACM in patients with AAV, which was comparable to conventional mortality risk factors in the general population.^9^ However, our previous study had the following issues: first, the sample size was insufficient to generalise and immediately apply the results to patients with AAV; second, the follow-up was not sufficiently long to fully evaluate subsequent serious complications of AAV; and third, subsequent serious complications did not include wide-ranging poor outcomes, such as relapse, ESKD, CVA, and ACS, but only ACM.^9^ To overcome these issues, we analysed data from 319 patients with AAV who had relatively long follow-up durations. We also investigated the predictive ability of baseline CAR for ACM, relapse, ESKD, CVA, and ACS in patients with AAV.

## Methods

### Patients

In this study, we screened data from 344 patients diagnosed with AAV, enrolled in the Severance Hospital ANCA-associated VasculitidEs (SHAVE) cohort, an observational cohort of Korean patients with AAV. The inclusion criteria were as follows: i) First classification/diagnosis of AAV was made in this hospital by two specialised rheumatologists between November 2005 and December 2025; ii) Fulfilment of the 2007 European Medicines Agency algorithm for AAV and polyarteritis nodos;a^2^ iii) Categorisation of AAV according to the 2012 revised Chapel Hill Consensus Conference Nomenclature of Systemic Vasculitide;s^1^ iv) Classification and/or reclassification of AAV proposed by a joint group of the American College of Rheumatology and the European Alliance of Associations for Rheumatology in 2022^10^, ^11^, ^12^, ^13^ v) Well-documented medical records sufficient to collect clinical, laboratory, radiological, and/or histological information for AAV diagnosis, both at the time of diagnosis and during the follow-up period; vi) Presence of ANCA test results within 4-weeks before and after AAV diagnosis; vii) Absence of concurrent medical conditions mimicking AAV, such as malignancy, serious infectious diseases, and systemic vasculitis other than AAV^10^^–^;^12^^,^^14^^,^^15^ viii) No exposure to immunosuppressive drugs for AAV treatment within 4-weeks before AAV diagnosis; and ix) Follow-up ≥3-months after AAV diagnosis. Of the 344 potentially eligible patients, 25 were excluded due to shorter follow-up; as such, data from 319 patients were included and analysed in this study.

### Ethics disclosure

The present study was approved by the Institutional Review Board (IRB) of Severance Hospital, Seoul, Republic of Korea (IRB n° 4–2016–0901), and was conducted in accordance with the principles of the Declaration of Helsinki. All patients included in this study provided written informed consent upon enrolment in the SHAVE cohort (i.e., at the time of AAV diagnosis and blood sampling). The IRB waived the requirements for additional written informed consent when it was obtained upon enrolment in the SHAVE cohort.

### Baseline clinical data

Baseline data refer to those recorded at the time of AAV diagnosis. Demographic data, including age, sex, Body Mass Index (BMI), and smoking history, were collected. AAV-related variables included AAV subtype, ANCA type and positivity and AAV-specific indices including the following: AAV subtype consisted of Microscopic Polyangiitis (MPA), Granulomatosis with Polyangiitis (GPA), and Eosinophilic GPA (EGPA)^10^^–^;^12^ ANCA type included Myeloperoxidase (MPO)-ANCA and Proteinase 3 (PR3)-ANCA measured by immunoassays as well as Perinuclear (P)-ANCA and Cytoplasmic (C)-ANCA detected by indirect immunofluorescence assays^16^; ANCA positivity was defined as positivity for at least one of these ANCA tests; and ANCA-specific indices comprised the Birmingham Vasculitis Activity Score (BVAS) and the Five-Factor Score (FFS).^17^^,^^18^ Type 2 Diabetes Mellitus (T2DM), hypertension, and dyslipidaemia were considered as baseline general comorbidities.^19^ Laboratory results, including Erythrocyte Sedimentation Rate (ESR), and CRP levels, were recorded.^20^ CAR was calculated as: CRP levels/serum albumin level.^9^

### Subsequent serious complications

Five subsequent serious complications were evaluated: ACM, relapse, ESKD, CVA, and ACS. ACM was defined as death from any aetiology, relapse as the development of at least one new manifestation of active vasculitis or the recurrence of a previous manifestation after remission had been maintained for at least 3-months,^21^ ESKD as a medical condition requiring renal replacement therapy,^22^ CVA as an ischaemic or haemorrhagic stroke confirmed by neurological evaluation and/or brain imaging,^23^^,^^24^ and ACS as acute myocardial infarction or unstable angina diagnosed on the basis of compatible clinical symptoms together with electrocardiographic changes and/or elevated cardiac biomarkers, with coronary angiographic findings considered when available.^25^ Among the five subsequent serious complications, ACM, ESKD, and CVA were investigated in this study. This was because relapse and ACS exhibited no statistical significance in a Receiver Operating Characteristic (ROC) curve analysis (Supplementary Fig. 1). The follow-up duration for each subsequent serious complication was calculated separately in the entire cohort and defined as the period from AAV diagnosis to its first occurrence for patients with the corresponding complication, whereas as the period from AAV diagnosis to the last visit or death, whichever occurred first, for patients without the corresponding complication.

### Medications for AAV treatment

Glucocorticoids and immunosuppressive drugs administered during follow-up were recorded. In this study, rituximab, cyclophosphamide, mycophenolate mofetil, azathioprine, tacrolimus, and methotrexate were investigated as immunosuppressive drugs.^26^^,^^27^

### Statistical analyses

All statistical analyses were performed using SPSS version 26 (IBM Corporation, Armonk, NY, USA) for Windows (Microsoft Corporation, Redmond, WA, USA). Continuous and categorical variables are expressed as medians (25−75 percentiles) and numbers (percentages). Correlation coefficients (*r*) between the two nonparametric variables were determined using the Spearman correlation analysis. A standardised correlation coefficient (β) was obtained by the multivariable linear regression analysis using variables with statistical significance in the univariable analysis. A significant Area Under the Curve (AUC) was determined using the ROC curve analysis. The cut-off for each subsequent serious complication was obtained by performing the ROC curve analyses and selecting the value with the maximised sum of sensitivity and specificity. The Relative Risk (RR) of the cut-off for each subsequent serious complication was analysed using contingency tables and the Chi-Square test. A comparison of the cumulative survival rates between the two groups was performed using the Kaplan-Meier survival analysis with the log-rank test. A multivariable Cox proportional hazard analysis with backward likelihood method using variables with p < 0.05 in a univariable Cox analysis was performed to calculate a Hazard Ratio (HR) during follow-up. For ESKD and CVA, Fine-Gray competing-risk regression analyses were additionally performed as sensitivity analyses, treating all-cause mortality as a competing event. Differences with p < 0.05 were considered to be statistically significant.

## Results

### Baseline clinical data

The median age of the 319 patients with AAV (64.6% female) was 60.0-years, of whom, 180 (56.4%), 77 (24.1%), and 62 (19.4%) had MPA, GPA, and EGPA, respectively, whereas 122 (38.2%), 58 (18.2%), and 46 (14.4%) had hypertension, T2DM, and dyslipidaemia, respectively. The detection frequencies of MPO-ANCA (or P-ANCA) and PR3-ANCA (or C-ANCA) were 70.8% and 15.4%, respectively. The median BVAS, FFS, ESR, and CRP levels were 12.0, 1.0, 58.5 mm/h, and 10.3 mg/L, respectively. The remaining laboratory investigation results are summarised in Table 1.

**Table 1:** Clinical data of patients with AAV.

**Variables**	**Values**
***At the time of AAV diagnosis***	
**Demographic data**	
Age (years)	60.0 (50.0 − 69.0)
Male sex, n (%)	113 (35.4)
Female sex, n (%)	206 (64.6)
BMI (kg/m^2^)	22.3 (20.2 − 24.6)
Ex-smoker	10 (3.1)
**AAV subtype, n (%)**	
MPA	180 (56.4)
GPA	77 (24.1)
EGPA	62 (19.4)
**ANCA type and positivity, n (%)**	
MPO-ANCA (or P-ANCA) positivity	226 (70.8)
PR3-ANCA (or C-ANCA) positivity	49 (15.4)
**AAV-specific indices**	
BVAS	12.0 (6.0 − 17.0)
FFS	1.0 (0 − 2.0)
**Comorbidities, n (%)**	
T2DM	58 (18.2)
Hypertension	122 (38.2)
Dyslipidaemia	46 (14.4)
**Acute phase reactants**	
ESR (mm/hr)	58.5 (19.8 − 97.3)
CRP (mg/L)	10.3 (1.5 − 60.3)
**Laboratory results**	
White blood cell count (/mm^3^)	8850.0 (6280.0 − 12,430.0)
Haemoglobin (g/dL)	11.6 (9.7 − 13.3)
Platelet count (× 1000/mm^3^)	298.0 (228.0 − 388.0)
Blood urea nitrogen (mg/dL)	17.4 (12.5 − 29.7)
Serum creatinine (mg/dL)	0.9 (0.7 − 1.6)
Serum total protein (g/dL)	6.8 (6.1 − 7.3)
Serum albumin (g/dL)	3.7 (3.2 − 4.3)
**CAR**	**2.66 (0.39−18.67)**
***During the follow-up period***	
**Poor outcome, n (%)**	
ACM	43 (13.5)
Relapse	89 (27.9)
ESKD	50 (15.7)
CVA	17 (5.3)
ACS	10 (3.1)
**Follow-up duration based on each poor outcome (months)**^a^	
ACM	67.4 (31.9 − 108.2)
Relapse	47.0 (18.1–88.3)
ESKD	59.6 (22.2 − 99.7)
CVA	65.4 (27.4 − 107.2)
ACS	66.5 (30.0–107.2)
**Medications**	
Glucocorticoids	301 (94.4)
Rituximab	68 (21.3)
Cyclophosphamide	169 (53.0)
Mycophenolate mofetil	89 (27.9)
Azathioprine	158 (49.5)
Tacrolimus	24 (7.5)
Methotrexate	51 (16.0)

Values are expressed as a median (25−75 percentile) or n (%).

a Follow-up duration was calculated separately for each outcome in the entire cohort. Patients were followed from AAV diagnosis to the first occurrence of the corresponding outcome and were censored at the last visit or death, whichever occurred first, if the outcome did not occur. AAV, ANCA-Associated Vasculitis; ACS, Acute Coronary Syndrome; ANCA, Antineutrophil Cytoplasmic Antibody; BMI, Body Mass Index; MPA, Microscopic Polyangiitis; GPA, Granulomatosis with Polyangiitis; EGPA, Eosinophilic Granulomatosis with Polyangiitis; MPO, Myeloperoxidase; P, Perinuclear; PR3, Proteinase-3; C, Cytoplasmic; BVAS, The Birmingham Vasculitis Activity Score; FFS, The Five-Factor Score; T2DM, Type 2 Diabetes Mellitus; ESR, Erythrocyte Sedimentation Rate; CRP, C-Reactive Protein; CAR, The C-reactive protein to serum Albumin Ratio; ACM, All-Cause Mortality; ESKD, End-Stage Kidney Disease; CVA, Cerebrovascular Accident.

### Follow-up clinical data

Of the 319 patients, 43 (13.5%) died after a median of 67.4-months, and 89 (27.9%) relapsed after a median of 47.0-months. 50 (15.7%), 17 (5.3%), and 10 (3.1%) developed ESKD, CVA, and ACS after a median of 59.6, 65.4, and 66.5 months, respectively. Glucocorticoids were administered to 301 (94.4%) patients. The most frequently administered immunosuppressive drug was cyclophosphamide (53.0%), followed by azathioprine (49.5%) and mycophenolate mofetil (27.9%) (Table 1).

### Nonparametric correlation analysis

Baseline CAR was significantly correlated with current age (*r* = 0.289), BVAS (*r* = 0.384), FFS (*r* = 0.272), and ESR (*r* = 0.701). Additionally, baseline CAR significantly correlated with both current blood urea nitrogen (*r* = 0.160) and serum creatinine (*r* = 0.185) levels (Fig. 1). Among the remaining laboratory parameters, baseline CAR was significantly correlated with white blood cell count, haemoglobin, platelet count, and serum protein levels (Supplementary Table 1).

**Fig. 1:**
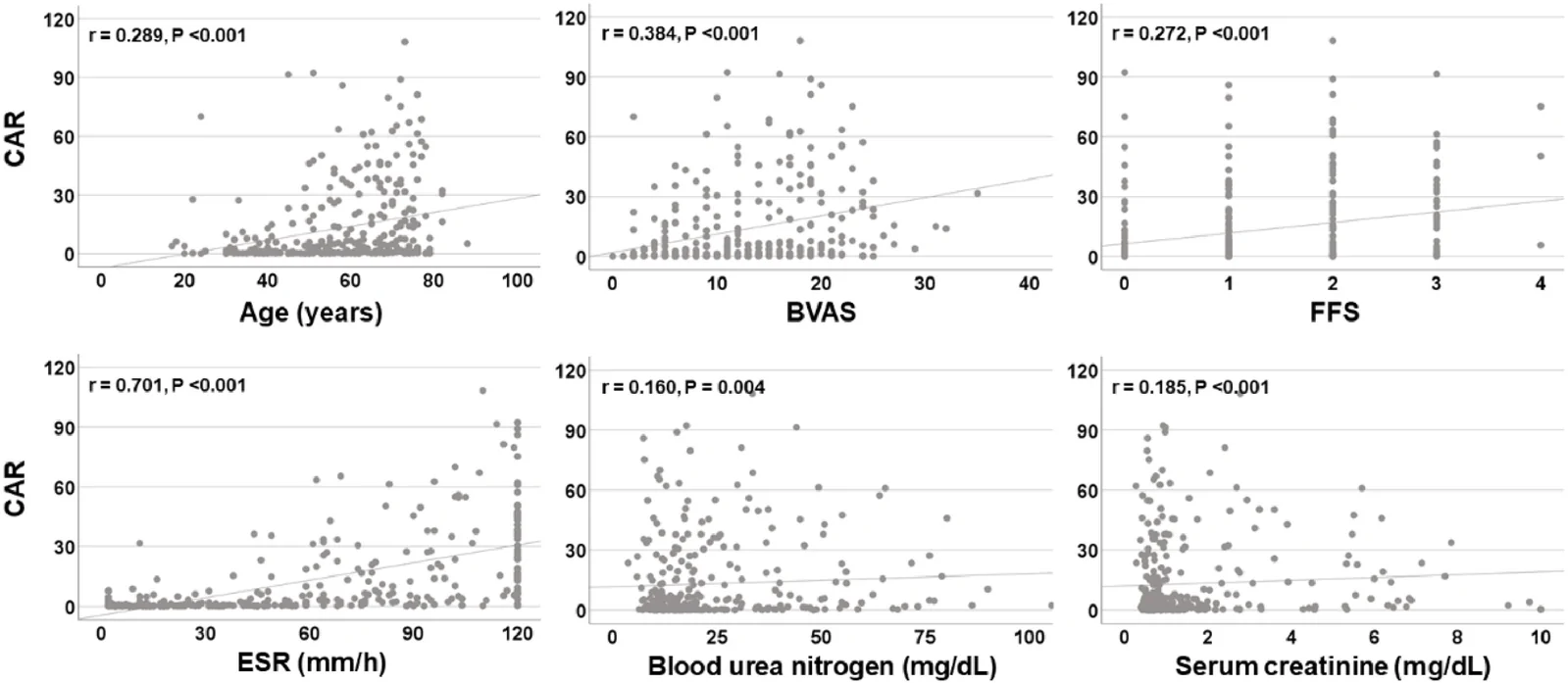
**Correlation analysis.** CAR, The C-reactive protein to serum Albumin Ratio; BVAS, the Birmingham Vasculitis Activity Score; FFS, The Five-Factor Score; ESR, Erythrocyte Sedimentation Rate.

### Linear regression for current BVAS

Univariable linear regression analysis revealed that baseline age, FFS, ESR, blood urea nitrogen, serum creatinine, and CAR were significantly correlated with current AAV activity, as assessed by BVAS. Multivariable analysis using these significant variables revealed that baseline CAR (standardised β = 0.167, 95% Confidence Interval [95% CI 0.014, 0.096]) was significantly and independently associated with current BVAS. Moreover, baseline FFS (standardised β = 0.228, 95% CI 0.679, 1.473), ESR (standardised β = 0.134, 95% CI 0.002, 0.042), and blood urea nitrogen (standardised β = 0.237, 95% CI 0.024, 0.130) were also significantly correlated with current BVAS but not serum creatinine levels (Table 2).

**Table 2:** Linear regression analysis of continuous variables for current BVAS in patients with AAV.

**Variables**	**Univariable**			**Multivariable**			
	**Beta**	**95% CI**	**p-value**	**Beta**	**95% CI**	**p-value**	**VIF**
Age (years)	0.127	0.008, 0.110	0.024	−0.091	−0.095, 0.010	0.114	1.307
BMI (kg/m^2^)	−0.046	−0.315, 0.132	0.419				
FFS	0.344	1.525, 2.844	<0.001	0.228	0.679, 1.473	<0.001	1.473
ESR (mm/hr)	0.275	0.028, 0.062	<0.001	0.134	0.002, 0.042	0.034	1.560
*CRP (mg/L)*	*0.302*	*0.024, 0.049*	*<0.001*				
Blood urea nitrogen (mg/dL)	0.300	0.063, 0.132	<0.001	0.237	0.024, 0.130	0.004	2.686
Serum creatinine (mg/dL)	0.236	0.477, 1.275	<0.001	−0.038	−0.731, 0.448	0.637	2.573
Serum total protein (g/dL)	−0.040	−0.244, 0.115	0.477				
*Serum albumin (g/dL)*	*−0.405*	*−4.582, −2.754*	*<0.001*				
CAR	0.298	0.062, 0.131	<0.001	0.167	0.014, 0.096	0.009	1.587

BVAS, Birmingham Vasculitis Activity Score; AAV, ANCA-Associated Vasculitis; ANCA, Antineutrophil Cytoplasmic Antibody; BMI, Body Mass Index; FFS, The Five-Factor Score; ESR, Erythrocyte Sedimentation Rate; CRP, C-Reactive Protein; CAR, The C-reactive protein to serum Albumin Ratio; VIF, Variance Inflation Factor.

### Comparison of AUCs for the highest tertile of BVAS among four baseline variables

In the ROC curve analysis, including the four baseline variables, that exhibited statistical significance in the multivariable linear regression analysis for current BVAS (i.e., FFS, ESR, blood urea nitrogen, and CAR), the AUCs for the highest tertile of BVAS (≥ 15) were compared. Among the four variables, baseline CAR yielded the numerically largest AUC for the highest tertile of BVAS (area 0.721, 95% CI 0.663, 0.778) (Fig. 2). Pairwise DeLong’s test revealed that the AUC of baseline CAR was significantly greater than those of ESR and blood urea nitrogen (p = 0.035 and p = 0.029, respectively), whereas the difference between baseline CAR and FFS was not statistically significant (p = 0.286).

**Fig. 2:**
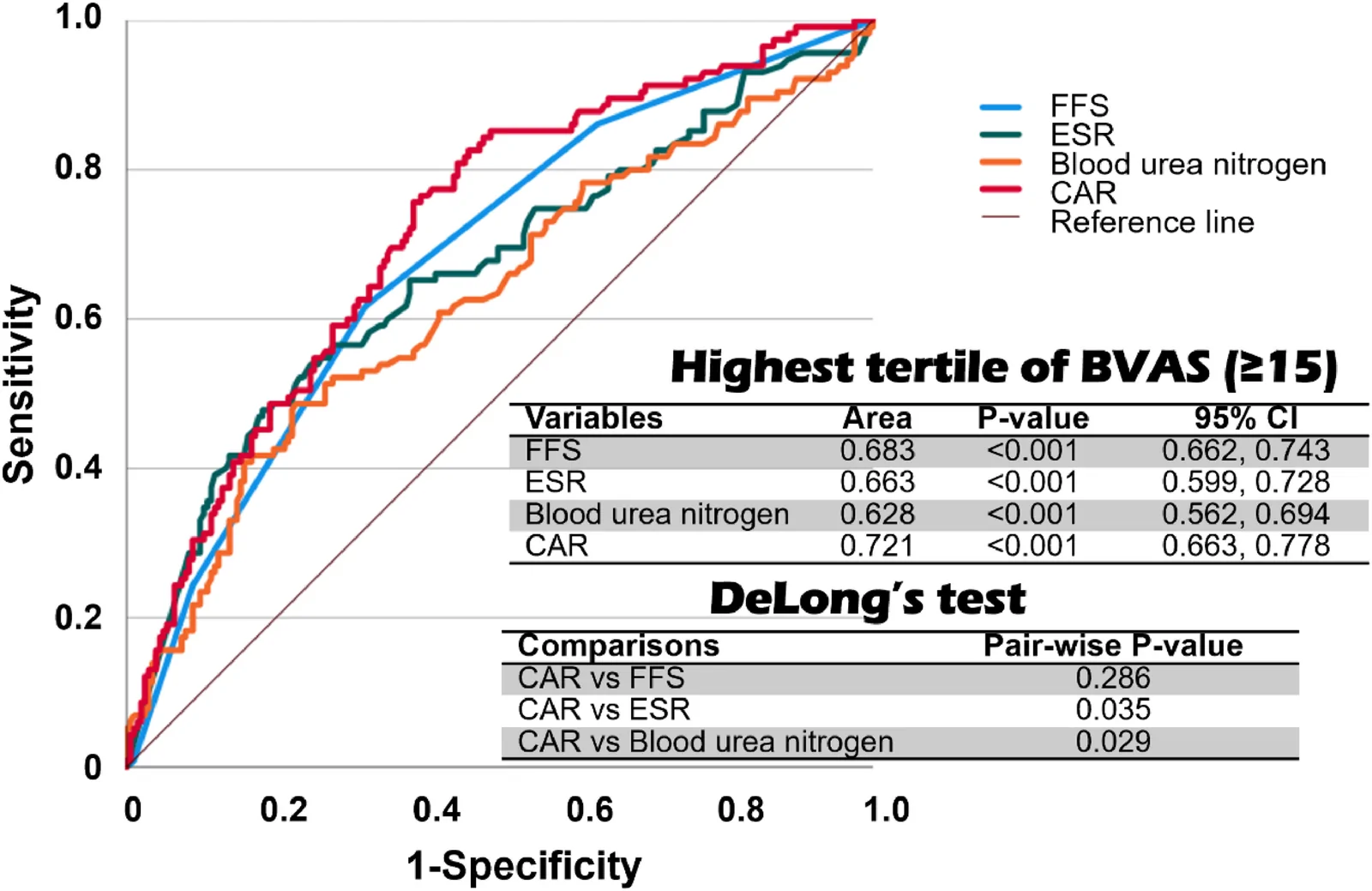
**Comparison of AUCs for the highest tertile of BVAS.** The AUCs were calculated using ROC curve analyses for identifying the highest tertile of BVAS, defined as BVAS ≥ 15. Baseline CAR showed the numerically largest AUC for the highest tertile of BVAS compared to FFS, ESR, and blood urea nitrogen. CAR, The C-reactive protein to serum Albumin Ratio; BVAS, The Birmingham Vasculitis Activity Score; FFS, The Five-Factor Score; ESR, Erythrocyte Sedimentation Rate.

### Cut-offs and relative risks of baseline CAR for subsequent ACM, ESKD, and CVA

ROC curve analysis revealed that baseline CAR exhibited statistically significant AUCs for ACM (area = 0.731, 95% CI 0.657, 0.805), ESKD (area = 0.643, 95% CI 0.568, 0.718), and CVA (area = 0.714, 95% CI 0.565, 0.863). Furthermore, the cut-off values of baseline CAR for ACM, ESKD, and CVA were determined to be 3.07 (sensitivity 86.0%, specificity 56.5%), 1.26 (sensitivity 80.0%, specificity 45.0%), and 14.01 (sensitivity 70.6%, specificity 73.5%), respectively (Fig. 3A and 3B). When patients were divided into two groups according to each cut-off, patients with baseline CAR ≥ 3.07, ≥ 1.26, and ≥ 14.01 exhibited significantly higher frequencies of ACM (23.6%vs. 3.7%, p < 0.001), ESKD (21.3%vs. 7.6%, p < 0.001), and CVA (13.0%vs. 2.2%, p < 0.001) than those for the corresponding opposite groups, respectively. Furthermore, the RRs for the corresponding subsequent complications were 8.017 for ACM (95% CI 3.276, 19.616), 3.270 for ESKD (95% CI 1.571, 3.977), and 6.660 for CVA (95% CI 2.275, 19.497) (Fig. 3B).

**Fig. 3:**
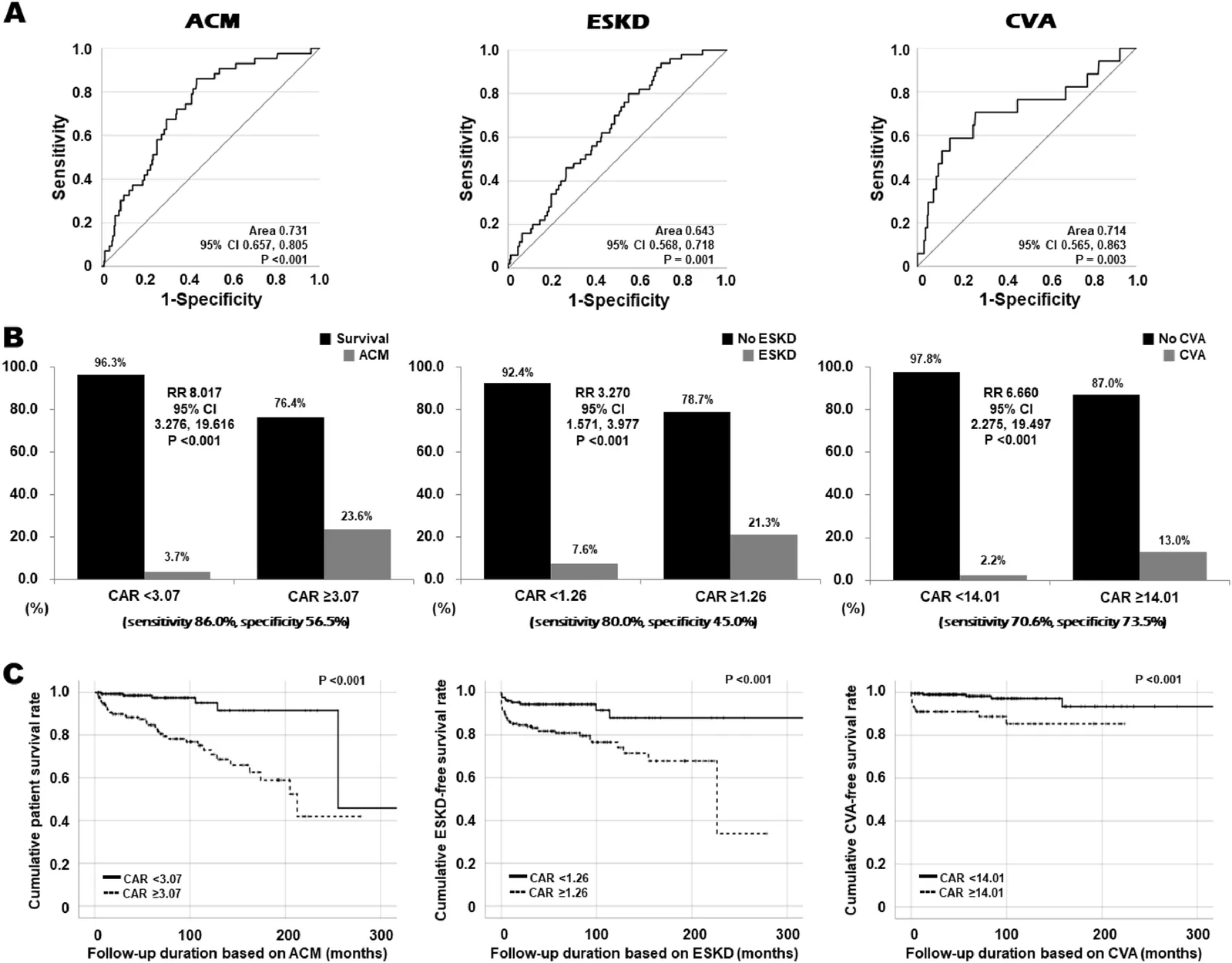
**Baseline CAR for subsequent ACM, ESKD, and CVA.** (A) ROC curve analysis for subsequent complications. (B) Comparison of RRs according to the cut-offs for the corresponding subsequent complications; and (C) Comparison of cumulative survival rates according to the cut-offs for the corresponding subsequent complications. CAR, The C-reactive protein to serum Albumin Ratio; ACM, All-Cause Mortality; ESKD, End-Stage Kidney Disease; CVA, Cerebrovascular Accident; ROC, Receiver Operating Characteristic; RR, Relative Risk; CI, Confidence Interval.

### Comparison of cumulative patient, ESKD-free, and CVA-free survival rates according to each cut-off for corresponding subsequent serious complications

Patients with baseline CAR ≥ 3.07, ≥ 1.26, and ≥ 14.01 yielded significantly reduced cumulative patient, ESKD-free, and CVA-free survival rates compared with each corresponding opposite group, respectively (p < 0.001 for all three variables) (Fig. 3C).

### Multivariable Cox proportional hazards analyses for subsequent ACM, ESKD, and CVA using the backward likelihood method

Results of the univariable Cox analyses are summarised in Supplementary Table 2. In the multivariable Cox analysis for ACM with either continuous CAR or with CAR ≥ 3.07, both baseline continuous CAR (HR = 1.018, 95% CI 1.007, 1.030) and CAR ≥ 3.07 (HR = 4.943, 95% CI 2.062, 11.851) were independently associated with subsequent ACM, respectively, alongside baseline age, serum creatinine, and rituximab use (Table 3A). In the multivariable Cox analysis for ESKD with CAR, baseline continuous CAR (HR = 1.017, 95% CI 1.004, 1.031) was independently associated with subsequent ESKD, together with baseline BVAS and serum creatinine; however, in the analysis with CAR ≥ 1.26, only baseline BVAS and serum creatinine, but not baseline CAR ≥ 1.26, were independently associated with subsequent ESKD (Table 3B). In the multivariable Cox analysis for CVA with either continuous CAR or with CAR ≥ 14.01, both baseline continuous CAR (HR = 1.023, 95% CI 1.006, 1.041) and CAR ≥ 14.01 (HR = 4.130, 95% CI 1.340, 12.731) were independently associated with subsequent CVA, respectively, along with baseline age, and rituximab use (Table 3C).

**Table 3:** Multivariable Cox proportional hazards analyses for ACM, ESKD, and CVA in patients with AAV using the backward likelihood method.

**A. ACM**			
**Step 8**	**Multivariable (with continuous CAR)**		
	**HR**	**95% CI**	**p-value**
Age (years)	1.070	1.037, 1.104	<0.001
Serum creatinine (mg/dL)	1.242	1.088, 1.418	0.001
Baseline continuous CAR	1.018	1.007, 1.030	0.002
Rituximab	3.854	2.000, 7.426	<0.001

	**Multivariable (with CAR ≥3.07)**		
	**HR**	**95% CI**	**p-value**
Age (years)	1.069	1.036, 1.104	<0.001
Serum creatinine (mg/dL)	1.223	1.069, 1.399	0.003
Baseline CAR ≥ 3.07	4.943	2.062, 11.851	<0.001
Rituximab	3.660	1.912, 7.007	<0.001

**B. ESKD**			
**Step 7**	**Multivariable (with continuous CAR)**		
	**HR**	**95% CI**	**p-value**
Age	1.023	0.998, 1.049	0.076
BVAS	1.057	1.013, 1.103	0.010
Hypertension	1.854	0.995, 3.457	0.052
Serum creatinine (mg/dL)	1.621	1.463, 1.796	<0.001
Baseline continuous CAR	1.017	1.004, 1.031	0.014

	**Multivariable (with CAR ≥ 1.26)**		
	**HR**	**95% CI**	**p-value**
BVAS	1.050	1.004, 1.097	0.033
FFS	1.401	0.992, 1.977	0.055
Hypertension	1.769	0.944, 3.313	0.075
Serum creatinine (mg/dL)	1.513	1.383, 1.655	<0.001
Baseline CAR ≥1.26	1.934	0.878, 4.259	0.102

**C. CVA**			
**Step 5**	**Multivariable (with continuous CAR)**		
	**HR**	**95% CI**	**p-value**
Age (years)	1.077	1.019, 1.138	0.009
Baseline continuous CAR	1.023	1.006, 1.041	0.010
Rituximab	5.329	1.849, 15.356	0.002

	**Multivariable (with CAR ≥ 14.01)**		
	**HR**	**95% CI**	**p-value**
Age (years)	1.072	1.015, 1.133	0.013
Baseline CAR ≥ 14.01	4.130	1.340, 12.731	0.014
Rituximab	6.810	2.329, 19.906	<0.001

ACM, All-Cause Mortality; ESKD, End-Stage Kidney Disease; CVA, Cerebrovascular Accident; CAR, The C-reactive protein to serum Albumin Ratio; HR, Hazard Ratio; CI, Confidence Interval; BVAS, The Birmingham Vasculitis Activity Score; FFS, The Five-Factor Score.

### Sensitivity analyses

The results of the sensitivity analyses were consistent with those of the primary Cox proportional hazards analyses. Baseline continuous CAR remained independently associated with both subsequent ESKD (subdistribution HR [sHR = 1.017], 95% CI 1.004, 1.031; p = 0.011) and CVA (sHR = 1.023, 95% CI 1.007, 1.039; p = 0.005). In addition, the ESKD-specific cut-off (CAR ≥ 1.26) remained non-significant (sHR = 1.633, 95% CI 0.721, 3.695; p = 0.239), whereas the CVA-specific cut-off (CAR ≥ 14.01) remained independently associated with subsequent CVA (sHR = 4.241, 95% CI 1.446, 12.444; p = 0.009) (Supplementary Table 3).

## Discussion

The present study included data from 319 patients with AAV who had relatively long follow-up to investigate the predictive ability of baseline CAR for ACM, relapse, ESKD, CVA, and ACS, and yielded several findings. First, baseline CAR was significantly correlated with current age, BVAS, FFS, ESR, blood urea nitrogen, and serum creatinine levels at diagnosis. Second, multivariable linear regression analysis revealed that, in addition to baseline FFS, ESR, and blood urea nitrogen levels, baseline CAR (standardised β = 0.167) was independently correlated with current BVAS. Third, ROC curve analysis revealed that baseline CAR yielded the numerically largest AUC for the highest tertile of BVAS (area = 0.721) compared with baseline FFS, ESR, and blood urea nitrogen levels; pairwise DeLong’s test showed that the AUC of baseline CAR was significantly greater than those of ESR and blood urea nitrogen, but not FFS.

Fourth, among the five subsequent complications, ROC curve analysis revealed significant AUCs for ACM, ESKD, and CVA. Fifth, patients with baseline CAR ≥ 3.07, ≥ 1.26, and ≥ 14.01 exhibited significantly higher risks for ACM (RR = 8.017), ESKD (RR = 3.270), and CVA (RR = 6.660) than the corresponding opposite groups, respectively. Sixth, patients with baseline CAR ≥ 3.07, ≥ 1.26, and ≥ 14.01 yielded significantly reduced cumulative patient, ESKD-free, and CVA-free survival rates compared with each corresponding opposite group, respectively. Seventh, multivariable Cox proportional analysis revealed that baseline CAR and its complication-related cut-offs were independently associated with subsequent ACM, ESKD, and CVA, except for baseline CAR ≥1.26 for ESKD. Accordingly, we concluded that baseline CAR has multipotent predictive ability; more specifically, it can not only reflect current high activity (i.e., the highest tertile of BVAS) but also predict subsequent ACM, ESKD, and CVA in patients with AAV.

In our previous study, which included 170 patients, baseline CAR was identified as an independent predictor of ACM, comparable with T2DM, a well-known conventional risk factor.^9^ The present study extended these previous findings by analysing a substantially larger cohort with a longer follow-up, thereby enhancing statistical power and the robustness of the results. Importantly, we confirmed that baseline CAR remained independently associated with ACM, reinforcing its reproducibility as a prognostic biomarker. Notably, the optimal cut-off value for predicting ACM was considerably lower in the current study (CAR = 3.07) than in a previous study (CAR = 10.35). This finding suggests that, compared with short-term ACM, even lower CAR values at diagnosis may be associated with long-term ACM, indicating that relatively modest elevation in CAR may have prognostic relevance over extended follow-up periods.

Beyond mortality, CAR has also been associated with other adverse outcomes, including acute kidney injury in patients with ST-segment elevation myocardial infarction undergoing primary percutaneous coronary intervention.^8^ The present study further expands the clinical implications of CAR by demonstrating its significant association with other major poor outcomes, including ESKD and CVA.^4^^,^^28^^,^^29^ A plausible explanation for these associations is that an elevated CAR reflects both increased systemic inflammation and reduced serum albumin levels. Systemic inflammation has been associated with endothelial dysfunction and platelet activation, which may contribute to CVA.^30^ In parallel, inflammation-related vascular and parenchymal injury may be associated with the progression of renal dysfunction.^31^ In addition, hypoalbuminaemia may reflect systemic inflammatory burden, malnutrition, or underlying organ dysfunction, all of which may be linked to adverse renal and vascular outcomes.^32^ Nevertheless, these mechanisms remain hypothetical, and the present observational study demonstrates associations only and cannot establish a causal relationship between CAR and the development of ESKD or CVA. These findings indicate that CAR is not merely a predictor of survival but also reflects the risk of important organ-specific complications in AAV. Given that these outcomes represent substantial contributors to morbidity and healthcare burden, identifying patients at increased risk at the time of diagnosis is of considerable clinical importance.^33^^,^^34^ In this context, CAR may serve as a practical tool for early risk stratification, enabling clinicians to identify high-risk patients who may benefit from more intensive monitoring and individualised clinical assessment.

In addition, we evaluated, for the first time, the cross-sectional relationship between baseline CAR and disease activity in AAV. Disease activity in AAV is typically assessed using BVAS, which, although comprehensive, requires the integration of diverse clinical, laboratory, and imaging parameters and may be less practical in routine clinical settings. In contrast, CAR is a simple and readily obtainable laboratory index. In the present study, baseline CAR showed significant correlation with BVAS and remained independently associated with disease activity, suggesting that it may serve as a complementary laboratory marker for the rapid assessment of current disease activity. While CAR cannot replace established scoring systems, its simplicity and accessibility support its potential utility as a complementary tool for the rapid and pragmatic assessment of disease status in routine clinical practice. To further evaluate its complementary prognostic role, we compared models including BVAS and FFS alone with models additionally incorporating baseline continuous CAR (Supplementary Table 4). The addition of CAR improved model discrimination and fit for ACM and CVA, with increases in the C-index, significant likelihood-ratio tests, and lower Akaike information criterion values. For ESKD, the addition of CAR produced a modest numerical increase in the C-index, although the improvement in overall model performance was not statistically significant. These findings suggest that CAR may provide clinically relevant incremental prognostic information beyond BVAS and FFS for selected outcomes, while its value for ESKD may lie primarily in its independent association with risk rather than in a substantial improvement in model discrimination. Therefore, CAR could be regarded as a simple complementary biomarker that may enhance, but not replace, established clinical assessment instruments.

The present study has several key strengths. First, the use of a well-defined cohort of 319 patients from a single tertiary centre, combined with an extensive follow-up period, can warrant superior statistical power compared to a previous study. Second, rather than limiting our focus to a solitary endpoint, this study conducted a comprehensive assessment of diverse clinical outcomes, including relapse, ESKD, CVA, ACS, and ACM. Finally, this study discovered the clinical utility of baseline CAR in estimating the current high activity of AAV, which may be useful in real clinical practice.

This study has several limitations that warrant consideration.

First, the findings may lack generalisability and external validity as the data were drawn from a single Korean tertiary referral centre; in addition, the tertiary referral setting may have introduced referral bias. Therefore, further prospective multicentre validation studies involving more diverse cohorts are necessary before CAR can be recommended for routine clinical risk stratification. Second, the retrospective, observational nature of this study precludes the direct causal links. Thus, our results should be viewed as associations rather than definitive evidence of causality. Third, although the multivariable analyses were adjusted for relevant immunosuppressive drugs, residual treatment-related confounding cannot be completely excluded. Fourth, the low incidence of specific events, such as ESKD and CVA, may have limited the statistical power required to draw robust conclusions for these outcomes. In particular, the small number of CVA events may have reduced the stability of the Cox regression estimates and widened the CIs; therefore, the findings regarding CVA should be interpreted cautiously. Finally, because the proposed CAR cut-off values were derived from the same study cohort, they should be considered exploratory and require external validation before clinical implementation.

## Conclusion

The present study demonstrated that baseline CAR has the multipotent predictive ability to reflect current high activity (the highest tertile of BVAS) and predict subsequent ACM, ESKD, and CVA in patients with AAV. Given its simplicity, baseline CAR may serve as a practical biomarker for early risk stratification, helping identify high-risk patients who require closer monitoring.

## Authors’ contributions

Conceptualization, H.S., O.C.K., and S.W.L.; Methodology, H.S., O.C.K., J.C., L.E.L., J.W.H., and S.W.L.; Validation, L.E.L., and Y.B.P.; Writing-original draft preparation, H.S., O.C.K., and S.W.L.; Data curation, H.S., O.C.K., and S.W.L. All the authors have read and agreed with the published version of the manuscript.

## Ethics approval and consent to participate

The present study was approved by the Institutional Review Board (IRB) of Severance Hospital, Seoul, Republic of Korea (IRB n° 4–2016–0901), and was conducted in accordance with the principles of the Declaration of Helsinki. All patients included in this study provided written informed consent upon enrolment in the SHAVE cohort (i.e., at the time of AAV diagnosis and blood sampling). The IRB waived the requirements for additional written informed consent when it was obtained upon enrolment in the SHAVE cohort.

## Consent for publication

Not applicable.

## Declaration of generative AI and AI-assisted technologies in the writing process

Not used.

## Statements

This manuscript has been written in accordance with the STROBE statement.

## Funding

This study was funded by Eisai Korea Inc. Seoul, Republic of Korea (4-2024-0700), Yuhan Corporation, Seoul, Republic of Korea, as part of their “2024 Investigator Initiated Translation Research Program” (4-2025-0044), Samsung Bioepis Co., Ltd., Incheon, Republic of Korea (4-2025-1066), and Samil Pharmaceutical Co., Ltd., Seoul, Republic of Korea (4-2025-1087; LFN-26-I01). The funder was not involved in the study design, collection, analysis, interpretation of data, the writing of this article, or the decision to submit it for publication. Therefore, we disclose that these analyses were pre-specified and conducted independently.

## Declaration of competing interest

The authors declare no conflicts of interest.

## Data Availability

The dataset generated and analysed during the current study is available from the corresponding author on reasonable request.
