# Supplementary material for: Multipotent ability of the baseline C-reactive protein-to-serum Albumin Ratio (CAR) to assess current activity and predict subsequent complications of ANCA-associated vasculitis
Source: Clinics (Sao Paulo). 2026 Nov 5;81:101217. doi: 10.1016/j.clinsp.2026.101217 (PMC13760100; doi:10.1016/j.clinsp.2026.101217)
Supplement: Supplementary file 1 [file mmc1.docx]

**CLINICS-D-26-01057 _ Supplementary Material**

**Supplementary Table 1** Nonparametric correlation analysis of CAR with continuous baseline variables in patients with AAV.

| **Variables** | **Correlation coefficient (*r*)** | **p-value** |
| --- | --- | --- |
| **Demographic data** |  |  |
| Age (years) | 0.289 | <0.001 |
| BMI (kg/m^2^) | -0.034 | 0.546 |
| **AAV-specific indices** |  |  |
| BVAS | 0.384 | <0.001 |
| FFS | 0.272 | <0.001 |
| **Acute phase reactants** |  |  |
| ESR (mm/hr) | 0.701 | <0.001 |
| *CRP (mg/L)* | *0.996* | *<0.001* |
| **Laboratory results** |  |  |
| White blood cell count (/mm^3^) | 0.419 | <0.001 |
| Haemoglobin (g/dL) | -0.518 | <0.001 |
| Platelet count (× 1000/mm^3^) | 0.401 | <0.001 |
| Blood urea nitrogen (mg/dL) | 0.160 | 0.004 |
| Serum creatinine (mg/dL) | 0.185 | <0.001 |
| Serum total protein (g/dL) | -0.332 | <0.001 |
| *Serum albumin (g/dL)* | *−0.672* | *<0.001* |

CAR, The C-reactive protein to serum Albumin Ratio; AAV, ANCA-Associated Vasculitis; ANCA, Antineutrophil Cytoplasmic Antibody; BMI, Body Mass Index; BVAS, The Birmingham Vasculitis Activity Score; FFS, The Five-Factor Score; ESR, Erythrocyte Sedimentation Rate; CRP, C-Reactive Protein.

**Supplementary Table 2** Univariable Cox proportional hazards analyses for ACM, ESKD, and CVA in AAV patients

| **ACM** |  | | |
| --- | --- | --- | --- |
|  | **Univariable** | | |
|  | **HR** | **95% CI** | **p-value** |
| Age (years) | 1.067 | 1.037, 1.098 | <0.001 |
| Male sex, n (%) | 1.918 | 1.019, 3.506 | 0.034 |
| BMI (kg/m^2^) | 0.973 | 0.886, 1.068 | 0.559 |
| Ex-smoker, n (%) | 2.589 | 0.797, 8.414 | 0.114 |
| MPO-ANCA (or P-ANCA) | 1.684 | 0.839, 3.381 | 0.142 |
| PR3-ANCA (or C-ANCA) | 0.577 | 0.226, 1.472 | 0.250 |
| BVAS | 1.070 | 1.026, 1.115 | 0.002 |
| FFS | 1.855 | 1.374, 2.504 | <0.001 |
| T2DM | 1.002 | 0.463, 2.167 | 0.996 |
| Hypertension | 2.105 | 1.144, 3.873 | 0.017 |
| Dyslipidaemia | 1.778 | 0.850, 3.723 | 0.127 |
| ESR (mm/hr) | 1.012 | 1.004, 1.020 | 0.003 |
| *CRP (mg/L)* | *1.008* | *1.004, 1.013* | *<0.001* |
| Blood urea nitrogen (mg/dL) | 1.012 | 1.004, 1.020 | 0.002 |
| Serum creatinine (mg/dL) | 1.141 | 1.022, 1.273 | 0.019 |
| Serum total protein (g/dL) | 0.639 | 0.453, 0.901 | 0.011 |
| *Serum albumin (g/dL)* | *0.401* | *0.275, 0.613* | *<0.001* |
| CAR | 1.023 | 1.012, 1.033 | <0.001 |
| CAR ≥3.07 | 6.381 | 2.687, 15.149 | <0.001 |
| Glucocorticoids | 2.573 | 0.353, 18.778 | 0.351 |
| Rituximab | 2.738 | 1.461, 5.129 | 0.002 |
| Cyclophosphamide | 1.092 | 0.588, 2.030 | 0.780 |
| Mycophenolate mofetil | 1.044 | 0.531, 2.051 | 0.901 |
| Azathioprine | 0.586 | 0.314, 1.094 | 0.093 |
| Tacrolimus | 1.486 | 0.621, 3.556 | 0.373 |
| Methotrexate | 0.477 | 0.170, 1.338 | 0.160 |
| **ESKD** |  | | |
|  | **Univariable** | | |
|  | **HR** | **95% CI** | **p-value** |
| Age (years) | 1.002 | 1.000, 1.044 | 0.049 |
| Male sex, n (%) | 0.788 | 0.423, 1.468 | 0.453 |
| BMI (kg/m^2^) | 0.918 | 0.839, 1.005 | 0.065 |
| Ex-smoker, n (%) | 0.686 | 0.095, 4.970 | 0.709 |
| MPO-ANCA (or P-ANCA) | 2.717 | 1.252, 5.895 | 0.011 |
| PR3-ANCA (or C-ANCA) | 0.680 | 0.289, 1.600 | 0.377 |
| BVAS | 1.084 | 1.042, 1.127 | <0.001 |
| FFS | 1.939 | 1.474, 2.552 | <0.001 |
| T2DM | 0.836 | 0.391, 1.786 | 0.643 |
| Hypertension | 2.939 | 1.650, 5.237 | <0.001 |
| Dyslipidaemia | 0.525 | 0.189, 1.460 | 0.217 |
| ESR (mm/hr) | 1.008 | 1.001, 1.015 | 0.023 |
| *CRP (mg/L)* | *1.005* | *1.001, 1.009* | *0.014* |
| Blood urea nitrogen (mg/dL) | 1.028 | 1.022, 1.033 | <0.001 |
| Serum creatinine (mg/dL) | 1.499 | 1.390, 1.618 | <0.001 |
| Serum total protein (g/dL) | 1.023 | 0.974, 1.075 | 0.356 |
| *Serum albumin (g/dL)* | *0.506* | *0.351, 0.728* | *<0.001* |
| CAR | 1.016 | 1.005, 1.027 | <0.001 |
| CAR ≥1.26 | 3.414 | 1.654, 7.049 | <0.001 |
| Glucocorticoids | 3.036 | 0.418, 22.048 | 0.272 |
| Rituximab | 1.309 | 0.680, 2.519 | 0.420 |
| Cyclophosphamide | 1.529 | 0.848, 2.757 | 0.158 |
| Mycophenolate mofetil | 1.096 | 0.588, 2.045 | 0.773 |
| Azathioprine | 0.659 | 0.372, 1.169 | 0.154 |
| Tacrolimus | 3.412 | 1.699, 6.851 | <0.001 |
| Methotrexate | 0.094 | 0.013, 0.682 | 0.019 |
| **CVA** |  | | |
|  | **Univariable** | | |
|  | **HR** | **95% CI** | **p-value** |
| Age (years) | 1.079 | 1.025, 1.136 | 0.004 |
| Male sex, n (%) | 1.356 | 0.481, 3.820 | 0.565 |
| BMI (kg/m^2^) | 1.104 | 0.962, 1.267 | 0.158 |
| Ex-smoker, n (%) | 0.047 | 0.000, 14811.815 | 0.637 |
| MPO-ANCA (or P-ANCA) | 1.978 | 0.554, 7.064 | 0.293 |
| PR3-ANCA (or C-ANCA) | 0.770 | 0.174, 3.417 | 0.731 |
| BVAS | 1.075 | 1.003, 1.153 | 0.041 |
| FFS | 1.905 | 1.160, 3.127 | 0.011 |
| T2DM | 1.046 | 0.295, 3.713 | 0.944 |
| Hypertension | 1.115 | 0.396, 3.142 | 0.837 |
| Dyslipidaemia | 1.533 | 0.432, 5.437 | 0.508 |
| ESR (mm/hr) | 1.010 | 0.997, 1.023 | 0.116 |
| *CRP (mg/L)* | *1.010* | *1.004, 1.017* | *0.002* |
| Blood urea nitrogen (mg/dL) | 1.006 | 0.998, 1.024 | 0.504 |
| Serum creatinine (mg/dL) | 0.934 | 0.681, 1.282 | 0.674 |
| Serum total protein (g/dL) | 0.484 | 0.278, 0.844 | 0.011 |
| *Serum albumin (g/dL)* | *0.332* | *0.167, 0.663* | *0.002* |
| CAR | 1.032 | 1.015, 1.049 | <0.001 |
| CAR ≥14.01 | 5.327 | 1.820, 15.590 | 0.002 |
| Glucocorticoids | 21.897 | 0.001, 412457.259 | 0.539 |
| Rituximab | 4.586 | 1.660, 12.670 | 0.003 |
| Cyclophosphamide | 1.189 | 0.421, 3.356 | 0.744 |
| Mycophenolate mofetil | 0.674 | 0.190, 2.392 | 0.541 |
| Azathioprine | 0.833 | 0.301, 2.306 | 0.725 |
| Tacrolimus | 1.623 | 0.365, 7.220 | 0.525 |
| Methotrexate | 1.223 | 0.344, 4.344 | 0.756 |

ACM, All-Cause Mortality; ESKD, End-Stage Kidney Disease; CVA, Cerebrovascular Accident; AAV, ANCA-Associated Vasculitis; ANCA, Antineutrophil Cytoplasmic Antibody; HR, Hazard Ratio; CI, Confidence Interval; BMI, Body Mass Index; MPO, Myeloperoxidase; P, Perinuclear; PR3, Proteinase 3; C, Cytoplasmic; BVAS, The Birmingham Vasculitis Activity Score; FFS, The Five-Factor Score; T2DM, Type 2 Diabetes Mellitus; ESR, Erythrocyte Sedimentation Rate; CRP, C-Reactive Protein; CAR, The C-reactive protein to serum Albumin Ratio.

**Supplementary Table 3** Sensitivity analysis: Fine-Gray competing-risk regression analyses for ESKD and CVA, treating all-cause mortality as a competing event.

| **A. ESKD** | | | |
| --- | --- | --- | --- |
| **Step 8** | **Multivariable (with continuous CAR)** | | |
|  | **sHR** | **95% CI** | **p-value** |
| BVAS | 1.065 | 1.023, 1.110 | 0.002 |
| Hypertension | 2.041 | 1.133, 3.677 | 0.018 |
| Serum creatinine (mg/dL) | 1.573 | 1.435, 1.723 | <0.001 |
| Baseline continuous CAR | 1.017 | 1.004, 1.031 | 0.011 |
|  | **Multivariable (with CAR ≥ 1.26)** | | |
|  | **sHR** | **95% CI** | **p-value** |
| Age | 1.032 | 1.008, 1.055 | 0.008 |
| BVAS | 1.058 | 1.017, 1.101 | 0.006 |
| Serum creatinine (mg/dL) | 1.618 | 1.469, 1.781 | <0.001 |
| Baseline CAR ≥ 1.26 | 1.633 | 0.721, 3.695 | 0.239 |
| **B. CVA** | | | |
| **Step 4** | **Multivariable (with continuous CAR)** | | |
|  | **sHR** | **95% CI** | **p-value** |
| Age (years) | 1.061 | 1.011, 1.114 | 0.016 |
| Baseline continuous CAR | 1.023 | 1.007, 1.039 | 0.005 |
| Rituximab | 3.238 | 1.228, 8.541 | 0.018 |
|  | **Multivariable (with CAR ≥ 14.01)** | | |
|  | **sHR** | **95% CI** | **p-value** |
| Age (years) | 1.057 | 1.007, 1.110 | 0.025 |
| Baseline CAR ≥ 14.01 | 4.241 | 1.446, 12.444 | 0.009 |
| Rituximab | 3.906 | 1.502, 10.157 | 0.005 |

ESKD, End-stage Kidney Disease; CVA, Cerebrovascular Accident; CAR, The C-reactive protein to serum Albumin Ratio; sHR, Subdistribution Hazard Ratio; CI, Confidence Interval; BVAS, The Birmingham Vasculitis Activity Score.

**Supplementary Table 4** Incremental prognostic value of baseline CAR beyond BVAS and FFS for subsequent ACM, ESKD, and CVA.

| **Outcome** | **Model 1 C-index** | **Model 2 C-index** | **ΔC-index** | **Likelihood-ratio p-value** | **AIC: Model 1 → Model 2** |
| --- | --- | --- | --- | --- | --- |
| ACM | 0.719 | 0.746 | +0.027 | 0.011 | 407.43 → 402.97 |
| ESKD | 0.755 | 0.761 | +0.006 | 0.455 | 497.45 → 498.89 |
| CVA | 0.687 | 0.787 | +0.100 | 0.001 | 181.74 → 173.57 |

Model 1 included BVAS and FFS. Model 2 included BVAS, FFS, and baseline continuous CAR.

ACM, All-Cause Mortality; ESKD, End-Stage Kidney Disease; CVA, Cerebrovascular Accident; CAR, The C-reactive protein to serum Albumin Ratio; BVAS, The Birmingham Vasculitis Activity Score; FFS, The Five-Factor Score; AIC, Akaike Information Criterion.

**Supplementary Figure 1**
